# Perspectives on the metabolism of strigolactone rhizospheric signals

**DOI:** 10.3389/fpls.2022.1062107

**Published:** 2022-11-24

**Authors:** Jian You Wang, Justine Braguy, Guan-Ting Erica Chen, Muhammad Jamil, Aparna Balakrishna, Lamis Berqdar, Salim Al-Babili

**Affiliations:** ^1^ The BioActives Lab, Center for Desert Agriculture, King Abdullah University of Science and Technology, Thuwal, Saudi Arabia; ^2^ Plant Science Program, Biological and Environmental Science and Engineering Division, King Abdullah University of Science and Technology (KAUST), Thuwal, Saudi Arabia

**Keywords:** strigolactones, rice (Oryza sativa), rhizosphere, root exudate, striga, LC-MS/MS

## Abstract

Strigolactones (SLs) are a plant hormone regulating different processes in plant development and adjusting plant’s architecture to nutrition availability. Moreover, SLs are released by plants to communicate with beneficial fungi in the rhizosphere where they are, however, abused as chemical cues inducing seed germination of root parasitic weeds, e.g. *Striga* spp., and guiding them towards host plants in their vicinity. Based on their structure, SLs are divided into canonical and non-canonical SLs. In this perspective, we describe the metabolism of root-released SLs and SL pattern in rice *max1-900* mutants, which are affected in the biosynthesis of canonical SLs, and show the accumulation of two putative non-canonical SLs, CL+30 and CL+14. Using *max1-900* and SL-deficient *d17* rice mutants, we further investigated the metabolism of non-canonical SLs and their possible biological roles. Our results show that the presence and further metabolism of canonical and non-canonical SLs are particularly important for their role in rhizospheric interactions, such as that with root parasitic plants. Hence, we proposed that the root-released SLs are mainly responsible for rhizospheric communications and have low impact on plant architecture, which makes targeted manipulation of root-released SLs an option for rhizospheric engineering.

## Introduction

Plants utilize metabolites as spatial chemical signals to coordinate their physiological functions and respond to environmental stimuli and conditions, including nutrition availability in the soil and rhizospheric signals (Weng et al., 2021). Responses and adjustments of plants to rhizospheric stimuli involves exudation of specific metabolites, which represents a key mechanism that facilitates interactions with neighboring plants, microbes, and parasites, and contributes to plants’ survival under abiotic and biotic stress environments (Massalha et al., 2017; Pang et al., 2021; Weng et al., 2021). Many of these specific signals derive from secondary metabolic pathways, such as the breakdown of carotenoids that provides precursors for the evolutionarily-conserved plant hormones abscisic acid and strigolactones (SLs) (Al-Babili and Bouwmeester, 2015; Wang et al., 2021), as well as different signaling and regulatory molecules, such as zaxinone and anchorene (Jia et al., 2019; Wang et al., 2019). SL biosynthesis starts with the reversible isomerization of all-*trans*-β-carotene to 9-*cis*-β-carotene by isomerase DWARF27 (Alder et al., 2012; Abuauf et al, 2018). Successive cleavage and rearrangement reactions accomplished by the CAROTENOID CLEAVAGE DIOXYGENASES (CCDs, CCD7 and CCD8) lead to carlactone (CL), the central intermediate of SL biosynthesis (Alder et al., 2012; Bruno and Al-Babili, 2016; Bruno et al., 2017; Chen et al., 2022) and the substrate of cytochrome P450 monooxygenase (CYP) 711A family, *MORE AXILLARY GROWTH 1 (MAX1)* (Zhang et al., 2014) (**Figure S1**).

Although SLs are commonly recognized as shoot branching inhibitors (Umehara et al., 2008), we recently reported that the rice canonical SLs 4-deoxyorobanchol (4DO) and orobanchol, which are secreted by roots, are not tillering-inhibitory hormones but important rhizospheric signals (Ito et al., 2022). In line with this discovery, tomato *CYP722C* mutant did not exert high branches although its canonical SLs orobanchol and solanacol were undetectable in its root exudates (Wakabayashi et al., 2019). Indeed, canonical SLs were originally identified as crucial rhizospheric signals, particularly in phosphate deprived environments, for plants to successfully recruit arbuscular mycorrhizal (AM) fungi for establishing a symbiosis that provides themselves with nutrients (Al-Babili and Bouwmeester, 2015; Lanfranco et al., 2018; Fiorilli et al., 2019). However, SL signals, including canonical ones, can be hijacked and used as chemo-attractants by root parasite weeds, such as *Striga* and *Phelipanche* spp., to ensure the presence of a host in the vicinity of their seeds when they germinate, which is required for the survival of these obligate parasitic plants (Lanfranco et al., 2018; Fiorilli et al., 2019; Ogawa et al., 2022).

As SL biosynthesis and exudation are generally induced under nutrient-limited conditions, secreted SLs might serve as communicators between non-parasitic plants themselves as well as with other organisms in the rhizosphere. This plant-plant communication may provide an advantage to the secreting plant, allowing the occupation of the space they need to increase the chance of survival. To address this hypothesis, we re-investigated the SL profiles of the rice *Os900* knockout (KO) lines by LC-MS/MS and identified a new putative CL-derived, non-canonical SL (CL+14, oxo-CL) accumulated in their exudates and root tissues. We showed that oxo-CL, together with CL+30, are mobile SLs, as they were the only SLs detected in the shoot base (1 cm-long stem tissue corresponding to the area connecting the roots and the shoot) (Ito et al., 2022). Moreover, to better understand the roles of these CL-derivatives, we investigated SL metabolic dynamics – through SL uptake and release – by feeding the SL biosynthesis mutants *d17* and *d10* with the exudates collected from wild-type (WT) and *max1-900* mutants. We observed interesting SL metabolic conversions as well as changes in the capability to induce parasitic weed germination. Based on our results, we propose that root-released SLs function as regulatory cues in rhizosphere communications and hypothesize CL-derived molecules might regulate rice plant architectures, although their biosynthetic enzyme(s) are yet uncharacterized.

## Results and discussion

### Oxo-CL, a novel putative non-canonical OsMAX1-900 substrate, accumulates in *Os900*-knockout mutant lines

**Figure 1:**
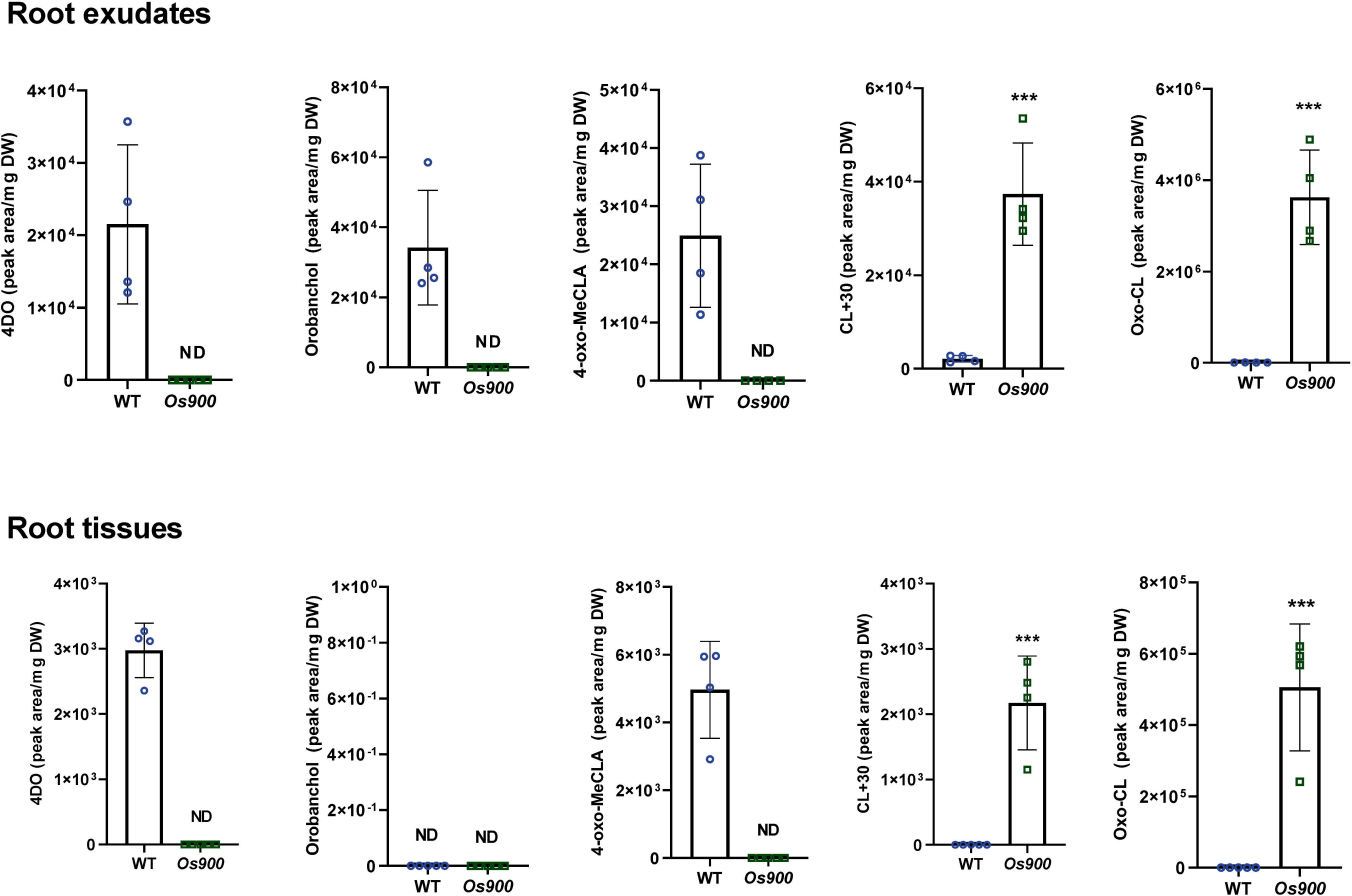
SL profile of WT and *max1-900* mutants grown under Low Pi conditions. Canonical and non-canonical SLs were quantified by LC-MS in root exudates and tissues. Bars represent mean ± SD; *n* = 4 biological replicates. Statistical analysis was performed using two-tail student t-test. Asterisks indicate statistically significant differences as compared to WT (***p< 0.001).. DW; dry weight.

Recently, we showed that *Os900* KO-mutant lines do not contain the canonical SLs 4DO and orobanchol, but accumulate a putative 4-oxo-hydroxyl-carlactone, called CL+30 (Ito et al., 2022). To get deeper insights into rice SL biosynthesis, we quantified the levels of SLs in root tissues and exudates of hydroponically grown *Os900* KO-mutant and WT plants under low Pi conditions, using Liquid Chromatography Tandem-Mass Spectrometry (LC–MS/MS) analysis. Our results confirmed the absence of 4DO and orobanchol in the *Os900*-KO lines, along with the accumulation of CL+30 (tentative namely 4-oxo-hydroxyl-carlactone) and the reduction in the putative 4-oxo-MeCLA (methyl 4-oxo-carlactonoate; also known as MeO-5DS isomer), in comparison to WT (**Figure 1**) (Ito et al., 2022). Moreover, we identified a novel putative SL molecule (CL + 14 Da, abbr. CL+14) that produced an ion peak characteristic of SL D-ring at *m*/*z* 97.02 and a parent ion at m/z 317.17 in root tissues and exudates of both *max1-900* lines (*Os900-*32 and -34) (**Figures S2-S3**). The molecular formula of CL+14 is C_19_H_24_O_4_ (*m*/*z* 317.17477 as positive ion [M + H]^+^, calculated for *m*/*z* 317.17474). To classify CL+14 as a SL and confirm that CL was its precursor, we fed *Os900-34* rice seedlings with [^13^C]-labeled CL. We identified [^13^C]-CL+14 in the root exudates, indicating this that metabolite is a downstream product of CL (**Figure S3**). However, we did not observe any significant change in the expression of SL biosynthetic genes in *Os900* mutants compared to the WT - under both +Pi and -Pi conditions (Ito et al., 2022) -, which might indicate that other unidentified enzyme(s), apart from MAX1 enzymes, can form CL-derivative(s).

Notably, CL+14 was also highly accumulated in *Os900* root exudates and tissues, in comparison to the WT (**Figure 1**). Moreover, we detected this compound in the root-shoot junctions, in addition to CL+30 (**Figure S4**), suggesting that these non-canonical SLs are mobile signals in rice (Ito et al., 2022) and are possible precursors of tillering/branching inhibitors. The significant accumulation of CL+14 in *Os900-*KO lines suggested that oxo-CL is another CL-derivative that acts as a substrate of OsMAX1-900. To validate this hypothesis, we treated WT plants with 10 μM TIS108, a MAX1 inhibitor (Ito et al., 2022), and analyzed the root exudates. Upon treatment, we observed an ~270-fold increase of this compound (**Figure S5**). These observations are in line with the analysis of SLs in *Os900*-KO line root exudates, indicating that CL+14 is a putative substrate for OsMAX1-900. Based on its mass difference to CL and the predicted chemical formula, CL+14 may include an oxo group at the C4 or C19 position, combined with the structure elucidation with MS/MS fragmentation (**Figure S3**), we therefore named this non-canonical SL oxo-CL. However, the exact chemical structure of oxo-CL requires further confirmation by Nuclear Magnetic Resonance (NMR).

### Metabolism of root released-SLs acts as communicating signals in rhizosphere

**Figure 2:**
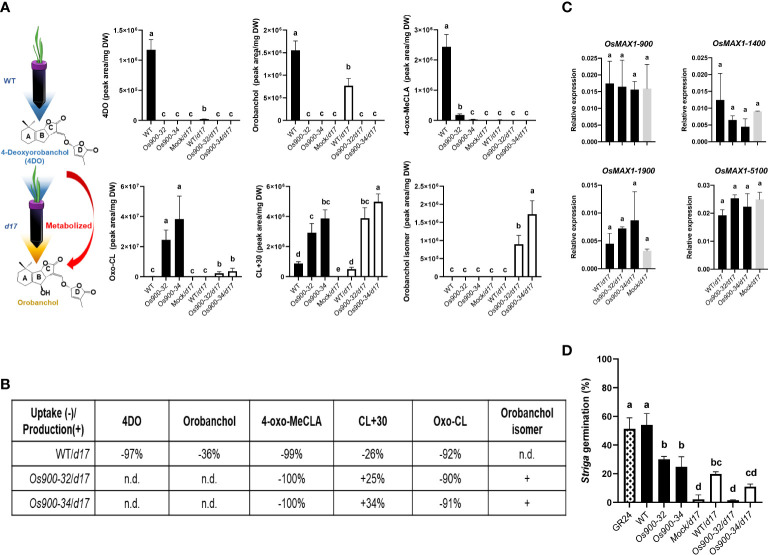
Feeding experiments and the metabolism of root-released SLs **(A)** Illustated scheme of feeding the expreriments and SLs quantification in the root exudates of WT, *Os900-32* and *-34* grown hydroponically under low Pi and used as feeding solution for the *d17*. 4-oxo-MeCLA (putative methyl 4-oxocarlactonoate), CL+30, 4DO (4-deoxyorobanchol), oxo-carlactone (oxo-CL), orobanchol isomer and orobanchol. The values are the mean ± SD for a number of 3-5 biological replicates. The statistical significance is determined by one-way ANOVA and Tukey’s multiple comparison test. **(B)** Percentage of SLs conversion: uptake **(-)** and production (+) by *d17* after being fed with readjusted (Pi and pH) exudates from WT, *Os900-32* and -34. Percentages indicate how much of the SLs present in the exudates were uptaken **(-)** by *d17* (and not released, like 4DO, orobanchol, 4-oxo-MeCLA isomer and oxo-CL) and produced/released (+) by the *d17* such as CL+30 and orobanchol isomer for *d17* plants fed with Os900 exudates. **(C)** Gene expression level of the four functional Nipponbare MAX1 genes in root tissues of 3-week old *d17* seedlings after feeding with WT (WT/*d17*) or *Os900-32*, *-34* (*Os900-32*/*d17 and Os900-34*/*d17*) root exudates, compared to mock (Mock/*d17*). The values are the mean ± SD number of biological replicates n (3 ≤ n ≤ 4). The statistical significance is determined by one-way ANOVA and Tukey’s multiple comparison test. **(D)** Striga germination assay after application of plant root exudates from the feeding experiment (Mock/*d17*, WT/*d17, Os900-32* and *-34/d17*) and the feeding solutions (WT, *Os900-32* and *-34)*. The values are the mean ± SD, number of biological replicates n (3 ≤ n ≤ 5). The statistical significance is determined by one-way ANOVA and Tukey’s multiple comparison test. DW; dry weight. Different letters denote significant differences (P < 0.05).

Several studies have reported that SL-deficient plants have higher number of branches/tillers and shorter shoots than WT (Al-Babili and Bouwmeester, 2015; Butt et al., 2018); however, the absence of 4DO and orobanchol did not result in high tillering in *Os900-*KO lines (Ito et al., 2022). This prompted us to study the role of CL+30 and oxo-CL in inhibiting tillering. To do so, high-branching *d17* plants were hydroponically grown and fed daily with root exudates of WT and *Os900* plants grown under low Pi condition (**Figure 2A**). After 3 weeks of treatment, the only noticeable phenotypical difference was the plant height: the fed plants were significantly higher with a longer second tiller than those under the mock condition, while no changes observed in the number of tillers (**Figure S6**). On the basis of this observation, we hypothesized the fed SLs were likely metabolized by MAX1 enzymes that are present in the *d17* mutant. To test this hypothesis, we analyzed root exudates and root tissues of fed *d17* plants (**Figure 2A**), and compared their SL pattern with that of WT and *Os900*, which were used for feeding (**Figure 2A**). We calculated the uptake based on the ratio between the contents in the exudate after feeding (fed) and the exudate used for feeding (unfed). We observed interesting uptake/release of different SLs by *d17*. When fed with the WT exudates, some of the released SLs were fully absorbed by *d17*, i.e. 92% of oxo-CL, 97% of 4DO and 99% of 4-oxo-MeCLA (**Figure 2B**). Others, such as orobanchol and CL+30, simply decreased partially in the fed exudates (36% and 26%, respectively). When fed with *Os900* exudates, 100% of 4-oxo-MeCLA and 90% of oxo-CL were absorbed by *d17* root, while CL+30 increased by 30%, as a mean of both *Os900* mutant lines (**Figure 2B**), suggesting that oxo-CL might be the precursor of CL+30. If this is the case, the oxo-CL detected here should be 4-oxo-CL (Ito et al., 2022). In addition, the level CL+30 seems to remain unchanged (**Figure 2A**), indicating a biological role of this metabolite in the rhizosphere. Hence, SLs released by plant roots are potentially communication signals between plants. Indeed, recent studies showed that canonical SLs released by rice and pea roots can affect the growth and development of nearby plants (Wheeldon et al., 2022; Yoneyama et al., 2022). Additionally, Yoneyama et al. (2022) demonstrated that co-planting of *d10* and WT rice seedlings suppressed the tillering phenotypes of *d10* (Yoneyama et al., 2022). However, we did not obtain the similar rescuing results, which could be due to the experimental setup or concentrations of SLs in root exudates. Nevertheless, these findings raise interesting questions on how plants sense, metabolize, and respond to different SLs in the soil.

Furthermore, we detected another interesting SL compound, containing the characteristic of SL D-ring at m/z 97.02, with the same m/z (347.140 as positive ion [M + H]^+^) as orobanchol (**Figure 2A**; **Figure S7**) but earlier retention time, which we named orobanchol isomer. Using Multiple Reaction Monitoring (MRM), we confirmed that the orobanchol isomer contained the same ion-pairs as orobanchol (**Figure S8**). Interestingly, orobanchol isomer was only detected in root exudates of *d17* fed with *Os900-32* and *-34* root exudates (**Figure 2A**), and its presence was also confirmed in WT root exudates once fed with *Os900-34* root exudates (**Figure S9**); however, we neither detected this compound nor orobanchol in root tissues of *d17* upon feeding with WT, *Os900-32* and *-34* root exudates (**Figure S10**). Gene expression analysis of *Os900*/*d17* root tissues did not show any changes in the SL biosynthetic genes (**Figure 2C**), which might be a hint for the involvement of yet unidentified enzyme(s) in the formation of CL+30 and orobanchol isomer and indicates that the SLs present in the exudates did not provoke significant negative feedback regulation loop. With respect to the production of orobanchol isomer, it seems that an isomerization step is necessary. To exclude the possibility that this isomer would come from a CL isomer produced by CCD8, we also fed *d10* (*ccd8* mutant) with *Os900-32* and *-34* root exudates. Orobanchol isomer was only detected by LC-MS/MS analysis when *d10* seedlings were fed with *Os900* KO-lines root exudates (**Figure S11**). Although orobanchol isomer might not exist naturally in WT rice plants, we proposed that SLs absorbed by roots are metabolized into orobanchol or a structurally similar compound.

As no differences were observed during the feeding experiment of *d17* with WT and *Os900* root exudates on tiller numbers and SL biosynthetic gene expression levels, we applied the resulting root exudates to test the biological activity of orobanchol isomer on *Striga seeds* germination. While the root exudates of WT, *Os900-32*, and *-34* were capable in inducing *Striga* seed germination, the exudates collected from *d17* plants after feeding with WT, *Os900-32* or *-34* exudates generally showed more than 50% reduction in the capability to induce *Striga* seeds germination, compared to root exudates of WT, *Os900-32*, and *-34* mutant lines (**Figure 2D**). This result suggests that root-released SLs were converted by plants into less potent *Striga* germination stimulants, such as orobanchol (**Figure 2D**). Intriguingly, the exudates of *Os900-34*/*d17* showed higher germination activity than *Os900-32*/*d17*, which might be due to the higher level of orobanchol isomer (**Figure 2D**). However, we did not observe this phenomenon with *P. ramosa* seeds (**Figure S12**), revealing that neither orobanchol nor orobanchol isomer were the preferred germinating signals for *P. ramosa*. Taken together, in addition to their role as strong stimulants of *Striga* seed germination and mycorrhization, canonical SLs may act as mobile signals in plant-plant and other plant-microbe communications.

### Conclusion and outlook

Canonical SLs do not play a major role in determining shoot architecture in rice, but are important signaling molecules in the rhizosphere (Ito et al., 2022). Indeed, root exudates of *Os900* mutants, which lack canonical SLs, have a lower activity in inducing germination in root parasitic seeds, compared to WT exudates. This reduction is not a result of a decrease in the total amount of released SLs, as the mutant lines have even more SLs in their exudates (**Figure 1**). However, it is likely a result of the absence of canonical SLs, especially 4DO, (**Figure 2**) which is a stronger seed germination stimulant than orobanchol (Ueno et al., 2011).

After the discovery of carlactone (CL) as a common intermediate in SL biosynthesis (Alder et al., 2012), the “real” shoot branching metabolite(s) and final product of plant SL biosynthesis remains elusive. Recently, we reported that the novel non-canonical SL CL+30 accumulated in *max1-900* rice mutants (Ito et al., 2022). In this work, we show that oxo-CL is also accumulated in the *max1-900* mutants, at even much higher level compared to CL+30. Although we cannot exclude the possibility that CL itself is the tiller inhibiting hormone, several studies reported on mutants showing a high-branching phenotype accompanied by CL accumulation (Abe et al., 2014; Brewer et al., 2016). Thus, the true branching-inhibitor in rice might be a CL-derivative(s), whose biosynthesis does not necessarily involve MAX1 enzymes.

In addition, the WT/*d17* feeding experiment showed that 97% of 4DO was absorbed by plants in the hydroponic system, while the uptake of orobanchol occurred at much lower level (**Figure 2B**). This indicates that orobanchol might be a final product in SL biosynthesis, which is released by roots and acts as an efficient rhizospheric signal. Indeed, orobanchol is the strongest stimulant of hyphal branching in AMF (Mori et al., 2016). Hence, knocking-out *MAX1-1400*, the likely final enzyme in the rice canonical SL biosynthesis (**Figure S1**) (Zhang et al., 2014), would be essential for complete elucidation of rice canonical SL biosynthesis. Moreover, the feeding experiment showed 90% uptake of oxo-CL and the production of CL+30 as well as orobanchol isomer by the *d17* mutant, indicating that oxo-CL may be a mobile signal between rice plants, which can be absorbed and converted into downstream metabolite(s) through the action of MAX1-900 and/or other CYPs (**Figure 2A**). Therefore, it is crucial to find out how rice plants transport SLs and to illuminate the metabolic capacity of MAX1 enzymes and the biological roles of canonical SLs in plants.

Although we did not observe a clear change in the tillering phenotype in the feeding experiments, further investigations by applying exudate containing higher amount of non-canonical SLs from natural source or organic synthesis to SL-deficient mutants will help for better understanding of the role of CL-derivatives (non-canonical SLs) in determining tillers number and plant’s height. Nevertheless, our data indicate that SLs released by plants do not have substantial effect on shoot growth and development. On the other hand, we demonstrate here that released SLs are important rhizosphere metabolites, and that their metabolism reduced the root exudates’ activity in inducing root parasitic seed germination. Hence, we hypothesize that rice plants have evolved metabolic pathways to modify particular SL structures for beneficial purpose, such as AM symbiosis. In addition, we propose an approach to investigate SL metabolism and probably SL signaling, by using root exudates as a feeding stock. Indeed, application of naturally accumulated SLs from SL-related mutants can be an alternative to treatment with SL chemically synthesized, expensive and in many cases commercially not vailable SLs.

Finally, the effect of a SL varies based on the stereochemistry and composition of SLs and is highly species-dependent; hence, feeding with root exudates can be a useful tool to investigate SL metabolism in different plant species and to depict similarities and differences in this process. For instance, it would very interesting to check if CL-derivatives discovered in rice can be metabolized by tomato plants? Or if 4DO can be metabolized by sorghum? If so, would these specialized metabolites serve as communication signals between plants? And how do plants distinguish SLs released by their neighbors from their own?

## Material and methods

### Plant material and growth conditions

*max1-900*, named here *Os900* (Ito et al., 2022), *d17* (Butt et al., 2018), and WT Nipponbare rice plants were grown under controlled conditions (a 12 h photoperiod, 200-µmol photons m^−2^ s^−1^ and day/night temperature of 27/25°C). Rice seeds were surface-sterilized in a 50% sodium hypochlorite solution with 0.01% Tween-20 for 15 min. Seeds were then rinsed with sterile water and germinated in the dark overnight. The pre-germinated seeds were transferred to Petri dishes containing half-strength liquid Murashige and Skoog (MS) medium and incubated in a growth chamber for 7 days. Thereafter, the seedlings were transferred into black falcon tubes filled with low-Pi of half-strength modified Hoagland nutrient solution with adjusted pH to 5.8. The nutrient solution consisted of 5.6 mM NH_4_NO_3_, 0.8 mM MgSO_4_·7H_2_O, 0.8 mM K_2_SO_4_, 0.18 mM FeSO_4_·7H_2_O, 0.18 mM Na_2_EDTA·2H_2_O, 1.6 mM CaCl_2_·2H_2_O, 0.8 mM KNO_3_, 0.023 mM H_3_BO_3_, 0.0045 mM MnCl_2_·4H_2_O, 0.0003 mM CuSO_4_·5H_2_O, 0.0015 mM ZnCl_2_, 0.0001 mM Na_2_MoO_4_·2H_2_O and 0.004 mM K_2_HPO_4_·2H_2_O.

### SL identification and quatificaiton in root exudate and tissues

SLs in rice root exudates were identified based on the protocol published in (Wang et al., 2022). In short, 50 mL of root exudates spiked with 0.672 ng of D_6_–5DS or 20 ng *rac*-GR24 was brought on a C_18_-Fast Reversed-Phase SPE column (500 mg 3 mL^-1^) preconditioned with 3 mL of methanol and 3 mL of water. After washing with 3 mL of water, SLs were eluted with 5 mL of acetone. The SL fraction was concentrated to SL aqueous solution (∼1 mL), followed by 1 mL of ethyl acetate extraction. 750 μL of SL enriched organic phase was dried under vacuum. For root tissues extraction, we followed the protocol described in (Wang et al., 2019). Around 50 mg of lyophilized and grinded *d17* rice root tissues, spiked with 0.672 ng of D_6_–5DS or 20 ng *rac*-GR24 as internal standards to control the extraction errors, were extracted twice with 2 mL of ethyl acetate in an ultrasound bath (Branson 3510 ultrasonic bath) for 15 min, followed by centrifugation for 8 min at 3800 rpm at 4°C. The two supernatants were combined and dried under vacuum. The residue was dissolved in 50 μL of ethyl acetate and 2 mL of hexane, following a Silica Cartridges SPE column (500 mg 3 mL^-1^) purification. After washing with 3 mL of hexane, SLs were eluted in 3 mL of ethyl acetate and evaporated to dryness under vacuum. The final extract was re-dissolved in 100 μL of acetonitrile: water (25:75, v:v) and filtered through a 0.22 μm filter for LC-MS/MS analysis.

The identification of SLs was performed by using UHPLC-Orbitrap ID-X Tribrid Mass Spectrometer (Thermo Scientific™) with a heated-electrospray ionization source. Chromatographic separation was achieved on the Hypersil GOLD C_18_ Selectivity HPLC Columns (150 × 4.6 mm; 3 μm; Thermo Scientific™) with mobile phases consisting of water (A) and acetonitrile (B), both containing 0.1% formic acid, and the following linear gradient (flow rate, 0.5 mL/min): 0–15 min, 25%–100% B, followed by washing with 100% B and equilibration with 25% B for 3 min. The injection volume was 10 μL, and the column temperature was maintained at 30 °C for each run. The MS conditions were as follows: positive mode, ion source of H-ESI, spray voltage of 3500V, sheath gas flow rate of 60 arbitrary units, auxiliary gas flow rate of 15 arbitrary units, sweep gas flow rate of 2 arbitrary units, ion transfer tube temperature of 350 °C, vaporizer temperature of 400 °C, S-lens RF level of 60, resolution of 120000 for MS; stepped HCD collision energies of 10 to 50% and resolution of 30000 for MS/MS. The mass accuracy of identified compounds (accurate mass ± 5 ppm mass tolerance) were acquired using Xcalibur software version 4.1.

SLs were quantified by LC-MS/MS using a UHPLC- Triple-Stage Quadrupole Mass Spectrometer (Thermo Scientific™ Altis™). Chromatographic separation was the same as above SL identification. The MS parameters were: positive ion mode, ion source of H-ESI, ion spray voltage of 5000 V, sheath gas of 40 arbitrary units, aux gas of 15 arbitrary units, sweep gas of 2 arbitrary units, ion transfer tube gas temperature of 350 °C, vaporizer temperature of 350 °C, collision energy of 17 eV, CID gas of 2 mTorr. The characteristic Multiple Reaction Monitoring (MRM) transitions (precursor ion → product ion) were 331.15→216.0, 331.15→234.1, 331.15→97.02 for 4-deoxyorobanchol; 347.14→329.14, 347.14→233.12, 347.14→ 205.12, 347.14→97.02 for orobanchol; 361.16→ 247.12, 361.16→177.05, 361.16→208.07, 361.16→97.02 for putative 4-oxo-MeCLA; 333.17→219.2, 333.17→173.2, 333.17→201.2, 333.17→97.02 for CL+30 (putative 4-oxo-hydroxyl-CL); 337.19→222.15, 337.19→240.16, 337.19→97.02 for D_6_-5-deoxystrigol. 299.09→158.06, 299.09→157.06, 299.09→97.02 for GR24. 317.17→ 220.14, 317.17→205.12, 317.17→164.08, 317.17→97.02 for CL+14 (putative oxo-CL).

### 13^C^-isotope feeding experiments

13^C^-CL was prepared following the protocol described previously (Bruno and Al-Babili, 2016). Around 20 ng 13^C^-CL was fed to two-week old *Os900* rice seedlings grown in low-Pi conditions for 6 h, and then 500 mL root exudates were collected for LC-MS/MS analysis.

### *d17* and *d10* feeding experiments using root exudates

*d17* and *d10* seeds were sterilized and germinated into Petri dish with +Pi solution. *d17* and *d10* seedlings were then transferred to 50 mL tubes (one plant per tube for phenotyping and two plants per tube for root exudate analysis experiment) containing the root exudates of two-weeks old WT*, Os900-32* or -*34* plants thatwere hydroponically grown two plants per 50 mL black tube under low Pi conditions. Pi content and pH of collected exudates were readjusted to 5.8 by the addition of K_2_HPO_4_.2H_2_O and HCl, before using them for feeding. Fresh low Pi solution was provided to the WT, *Os900-34* and -*34* seedlings daily; 6 h later, the exudates from the same genotype were pooled together, adjusted (Pi concentration was 0.4 mM) and fed to *d17* for two week.

For SL analysis, fresh low Pi solution was provided to the WT, *Os900-34* and -*34* seedlings daily; 24 h later, the exudates from the same genotype were pooled together, adjusted (Pi concentration was 0.4 mM) and fed to two week Pi starvation *d17* and *d10* seedlings for 6 h incubation; thereafter exudates were extracted according to Wang et al., 2022. The absorption/release ratios were calculated as [(fed exudate collected from *d* mutants)-(unfed exudate)/unfed exudate]*100

### Gene expression analysis

For transcript analysis, total RNA was extracted from rice roots using the Direct-zol RNA Miniprep Plus kit (Zymo research, #R2071), according to the manufacturer’s instructions. cDNA was synthetized from 2 µg of total RNA using iScript cDNA Synthesis Kit (BIO-RAD Laboratories, Inc, 2000 Alfred Nobel Drive, Hercules, CA; USA) according to the instructions in the user manual. qRT-PCR was performed using SYBR Green Master Mix (Applied Biosystems), which was diluted before use. Each PCR reaction was carried out in a total volume of 10 μL containing 4 μL diluted cDNA (the resulting cDNA was diluted 1:10 in H_2_O), 5 μL 2X diluted SYBR Green Reaction Mix, and 0.5 μL of each primer (2 μM working stock). All reactions were performed on a 384-well plate in a CFX384 Touch Real-Time PCR Detection System (Bio-Rad) as follows: 95°C for 90 sec, 40 cycles of 95°C for 15 sec, 60°C for 30 sec. All reactions were performed on at least three biological and three technical replicates, including a water control to exclude potential unspecific amplification. The 2^-ΔΔCT^ method was used to calculate the relative gene expression levels (Livak and Schmittgen, 2001) and rice Ubiquitin (UbiQ) gene was used as the internal control to normalize target gene expression (see **Table S1** for primer sequences).

### Striga hermonthica and Phelipanche ramosa seed germination bioassays

*Striga hermonthica* and *Phelipanche ramosa* seeds were first sterilized and pre-conditioned as described (Radoslava et al., 2005; Wang et al., 2022). Briefly, *S. hermonthica* seeds were sterilized with 20% bleach and 0.1% tween-20 for 5 min, while *P. ramosa* required 3 min wash with 75% EtOH, followed by 6 washes with sterile water and 3 min sterilization with 3% bleach. Both seeds species were washed 6 times with water before being dried for 3-4 hours under the flow of a laminar hood. Seeds were then spread on glass fiber discs before being preconditioned: at 30°C for 10 days for *Striga*, and 22°C for 14 days for *Phelipanche*. After application of the extracted root exudates, 50 μL for *Striga* and 100 μL for *Pheliphanche*, seeds were let to germinate for 24h at 30°C and 7 days at 25°C, respectively. Germinated (seeds with radicle) and non-germinated seeds were counted under a binocular microscope to calculate germination rate (%) by using the open-source SeedQuant software (Braguy et al., 2021.

### Statistical analysis

Data are represented as mean and their variations as standard deviation. The statistical significance was determined by one-way analysis of variance (one-way ANOVA) with Tukey’s multiple comparison test, using a probability level of p<0.05; or two-tail student t-test with denote significant differences (*p < 0.05, **p< 0.01, ***p< 0.001, ****p< 0.0001). All statistical elaborations were performed using GraphPad Prism, version 8.3.0.

## Data availability statement

All data generated or analyzed during this study are included in this published article.

## Author contributions

SA-B and JW proposed the concept and designed the experiments. JW, JB, G-TC, MJ, AB, and LB conducted experiments. JW, JB, and SA-B analyzed and discussed the data. JW, JB, G-TC, MJ, and SA-B read, edited, and approved the manuscript. All authors contributed to the article and approved the submitted version.

## Funding

This work was supported by baseline funding from King Abdullah University of Science and Technology and by the Bill & Melinda Gates Foundation (grant number OPP1136424) given to SA-B.

## Acknowledgments

We sincerely thank the support from the members of KAUST Analytical Core Lab and the Bioactives lab. We thank Dr. Abdel Gabbar Babiker for providing *Striga hermonthica* seeds.

## Conflict of interest

The authors declare that the research was conducted in the absence of any commercial or financial relationships that could be construed as a potential conflict of interest.

## Publisher’s note

All claims expressed in this article are solely those of the authors and do not necessarily represent those of their affiliated organizations, or those of the publisher, the editors and the reviewers. Any product that may be evaluated in this article, or claim that may be made by its manufacturer, is not guaranteed or endorsed by the publisher.
