# Supplementary material for: Perspectives on the metabolism of strigolactone rhizospheric signals
Source: Front Plant Sci. 2022 Nov 24;13:1062107. doi: 10.3389/fpls.2022.1062107 (PMC9729874; doi:10.3389/fpls.2022.1062107)
Supplement: Supplementary file 1 [file DataSheet_1.pdf]

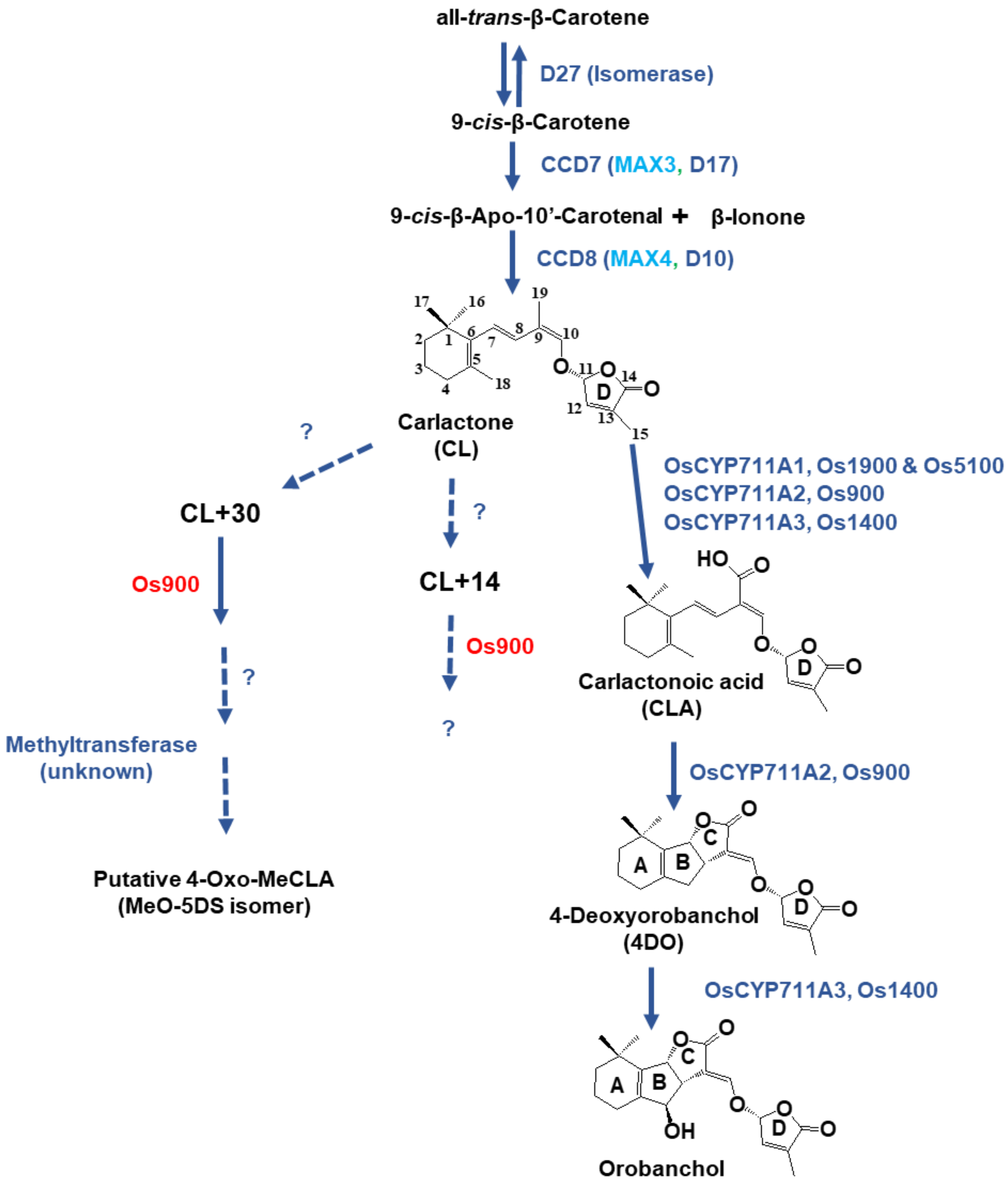

Supplementary Figure 1. SL biosynthetic pathway in rice.

Abbreviations: D27, Dwarf27; CCD, Carotenoid Cleavage Dioxygenase; MAX1, More Axillary Growth 1; CYP, Cytochrome P450; 5DS, 5-Deoxy-Strigol.

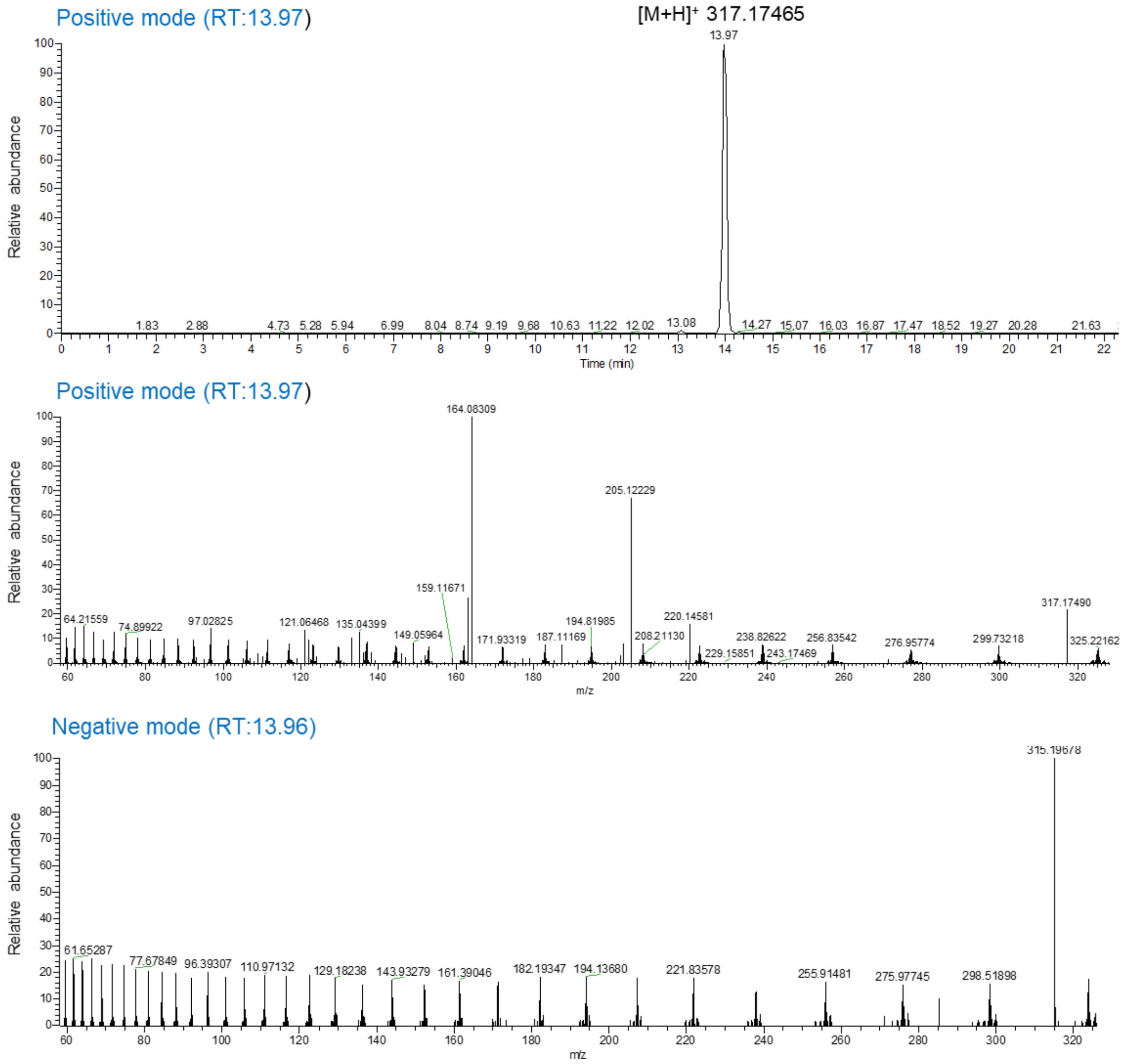

Supplementary Figure 2. The identification of the putative oxo-CL: LC-MS analysis of oxo-CL (Retention time: 13.97). Product ion spectra derived from the precursor ion (m/z 317.17465 $[M+H]^+$ in positive mode) and the precursor ion (m/z 315.19678 $[M-H]^-$ in negative mode) of CL + 14 Da in *Os900* exudate.

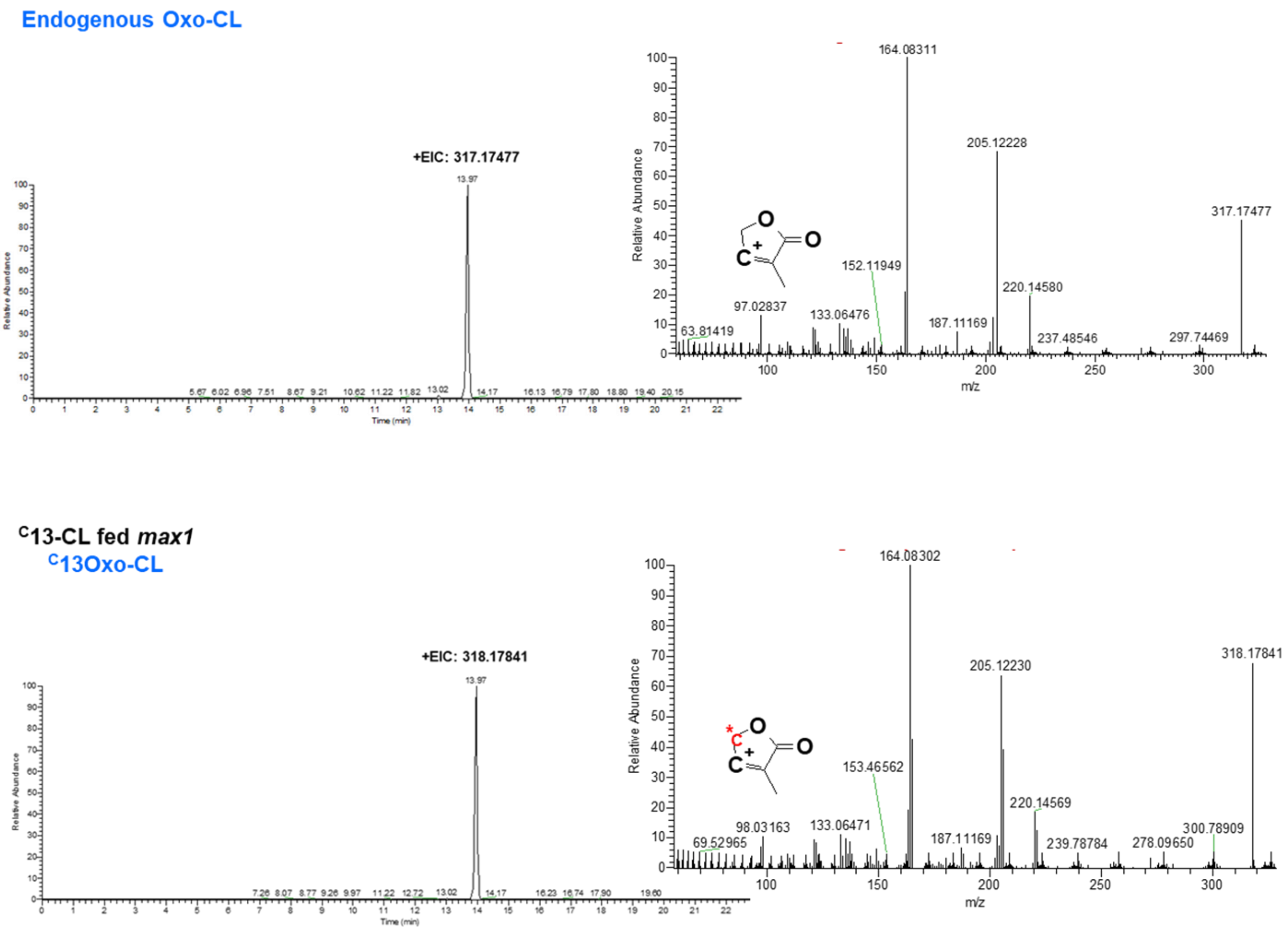

Supplementary Figure 3. EIC chromatography for the identification of endogenous oxo-CL. Product ion (Retention time: 13.97) spectra derived from the precursor ion (m/z 317.17477 $[M+H]^+$ in positive mode) with characterized D-ring at m/z 317.17477 > 97.02834. $[^{13}C]$-oxo-CL was characterized with ions pairs at m/z 318.17841 > 98.03163. The proposed structures of fragments are inserted.

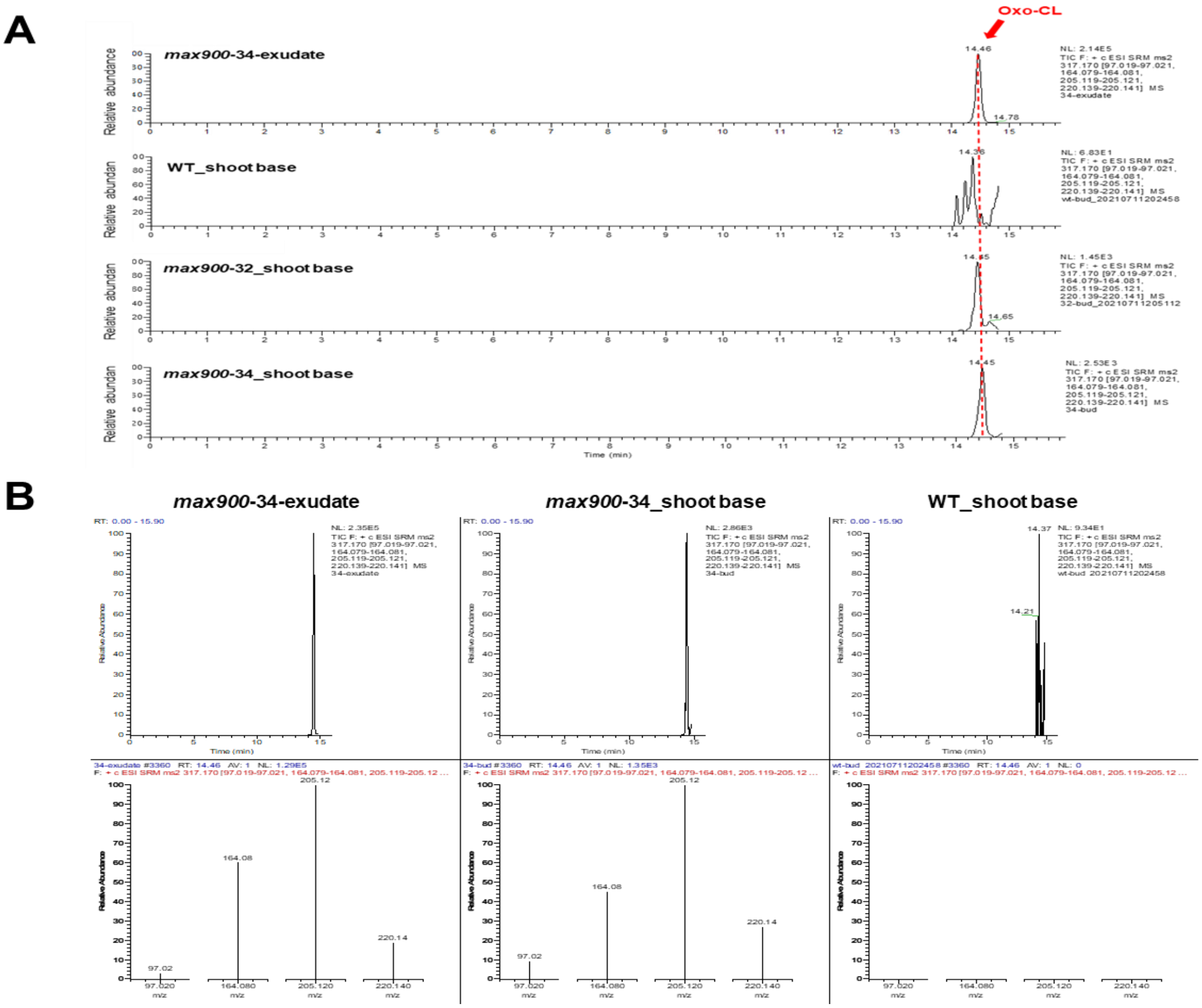

Supplementary Figure 4. Detection of oxo-CL in *Os900-34* root exudate and in the WT, *Os900-32* and -*34* shoot bases (low Pi condition). (A) From top to bottom: Chromatograms for oxo-CL detection in *Os900-34* root exudate, and WT, *Os900-32* and *-34* shoot base (stem tissue corresponding to the shoot-root junction of the seedling) of plants grown in low Pi condition. Oxo-CL was detected in *Os900-34* root exudate, as well as in *Os900-32* and *-34*'s shoot base; however, oxo-CL was not detected in the shoot bases of WT plants. (**B**) Representative oxo-CL identification by multiple reaction monitoring (MRM) of four m/z fragmentations with the diagnostic m/z 97.02 (D-ring). The shoot base extraction samples were obtained from a pool of 12 shoot bases of each genotype grown in low Pi condition. The red arrows indicate the elution peaks of oxo-CL with a retention time of 14.46 min.

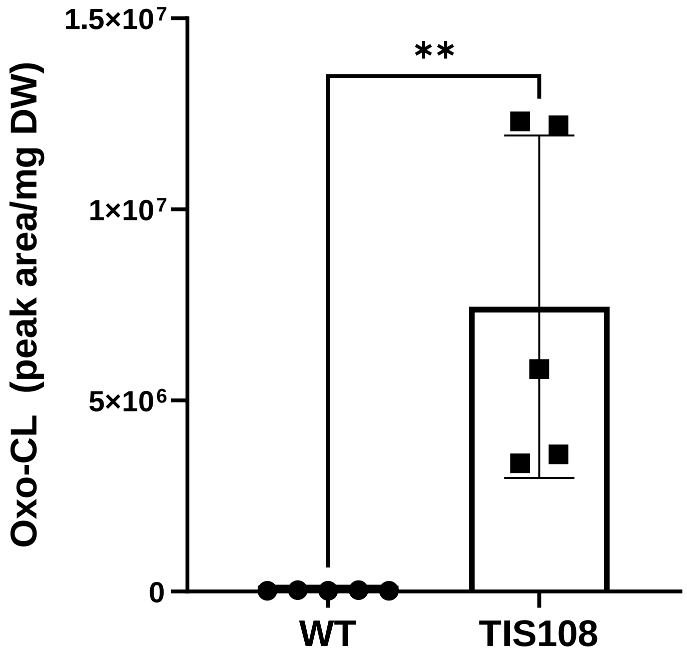

Supplementary Figure 5. Comparison of oxo-CL content in the root exudate contents upon TIS108 (10 μM) treatment in WT seedlings. The data are represented as the mean ± SD for a number of 5 replicates. Asterisk indicates significant difference without (Mock) and with 10 μM TIS108 treatment (TIS108) (**P<0.01, ***P<0.001, ****P≤0.0001, Student's t test).

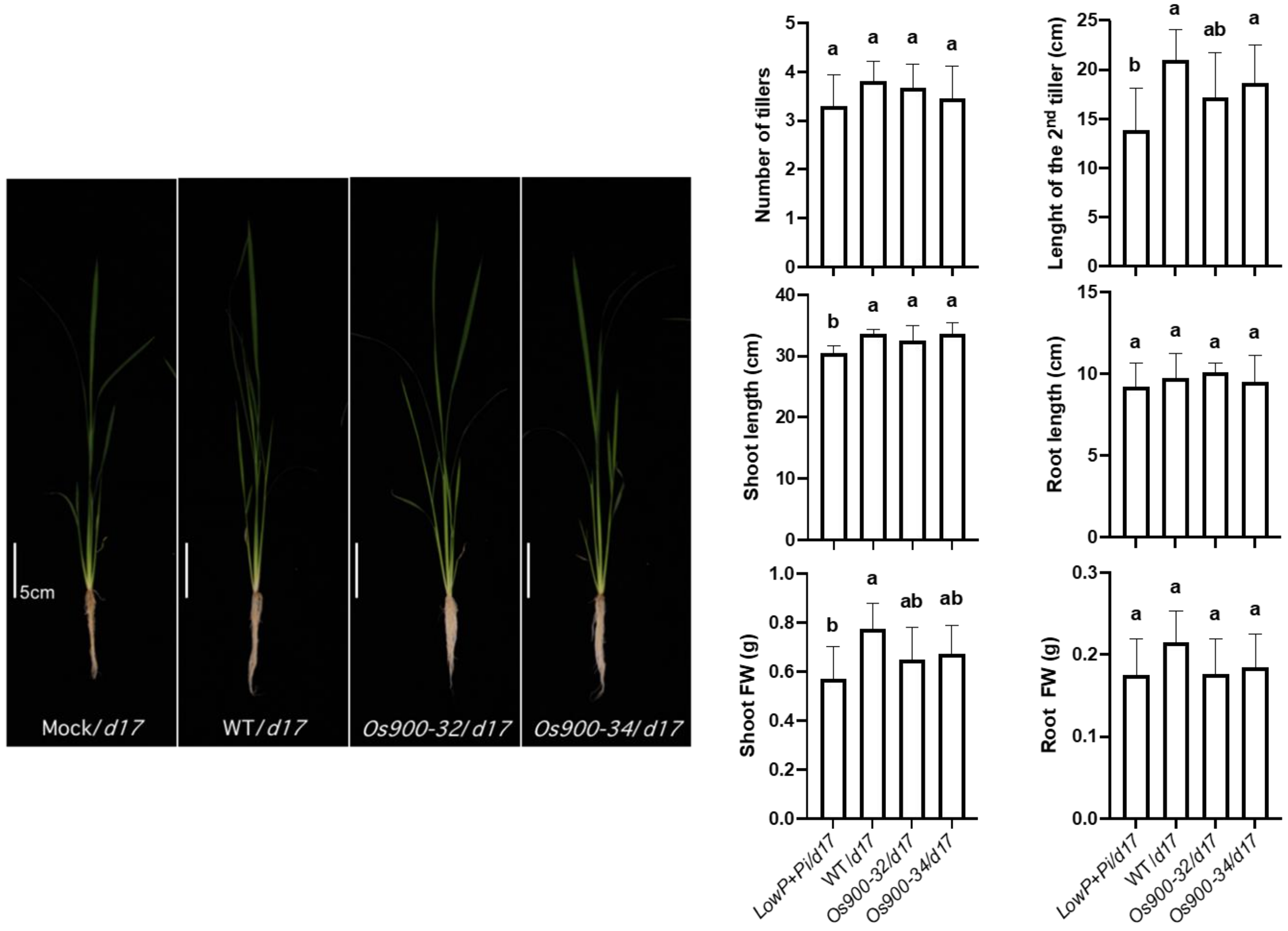

Supplementary Figure 6. *d17* feeding experiment with WT and *Os900*-KO root exudates revealed the presence of oxo-CL and orobanchol isomer in fed *d17* root exudates. Phenotype of the *d17* plants after hydroponic feeding experiment using mock (LowP+Pi/*d17;* Mock), WT (WT/*d17*) and *Os900*-KO lines (*Os900-32*/*d17 and Os900-34*/*d17*) root exudates. Scale bars = 5cm. The data are represented as the mean ± SD for a number n of replicates (10 ≤ n ≤ 13). The statistical significance is determined by one-way ANOVA and Tukey's multiple comparison test.

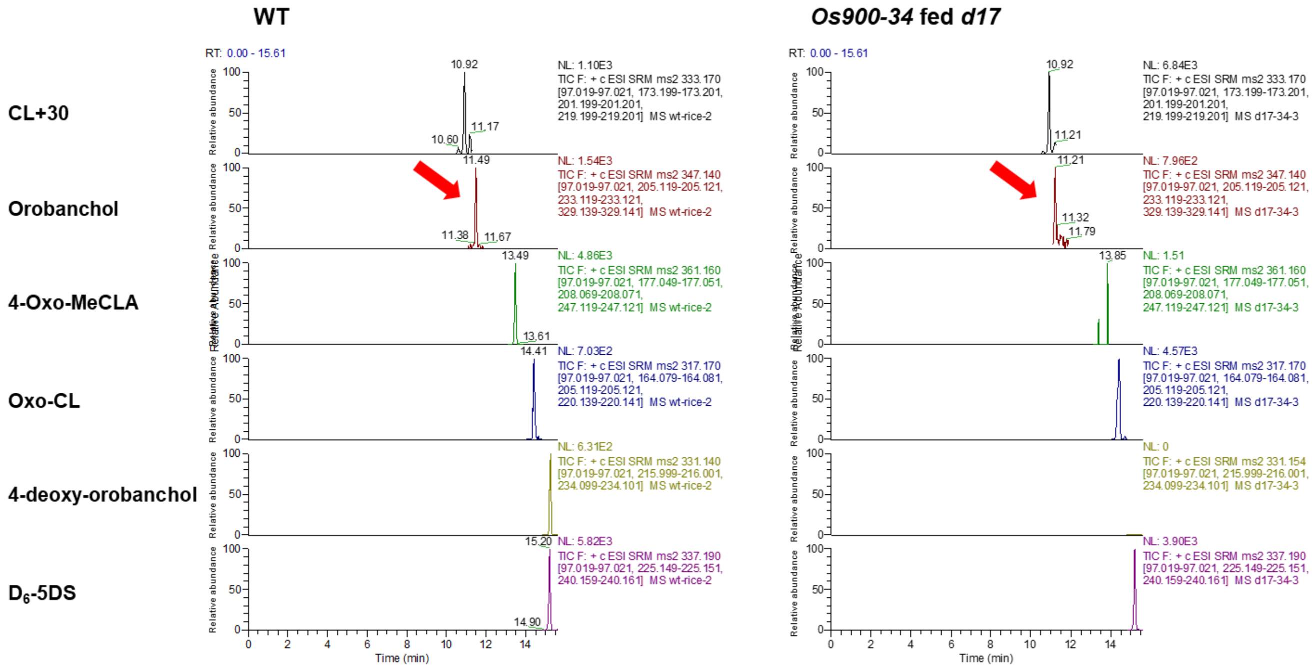

Supplementary Figure 7. SL quantification of *d17* feeding experiment with WT and *Os900*-KO. Chromatograms of the different SLs in WT and *d17* seedlings fed with *Os900-34* root exudates. Multiple reaction monitoring (MRM) was used to quantify SL level in WT and of *d17* root exudates, the later fed with readjusted (Pi and pH) *Os900-34* root exudates under low Pi grown conditions. Red arrows indicate the elution peaks of orobanchol with a retention time of 11.49 min or orobanchol isomer with a retention time of 11.21 min.

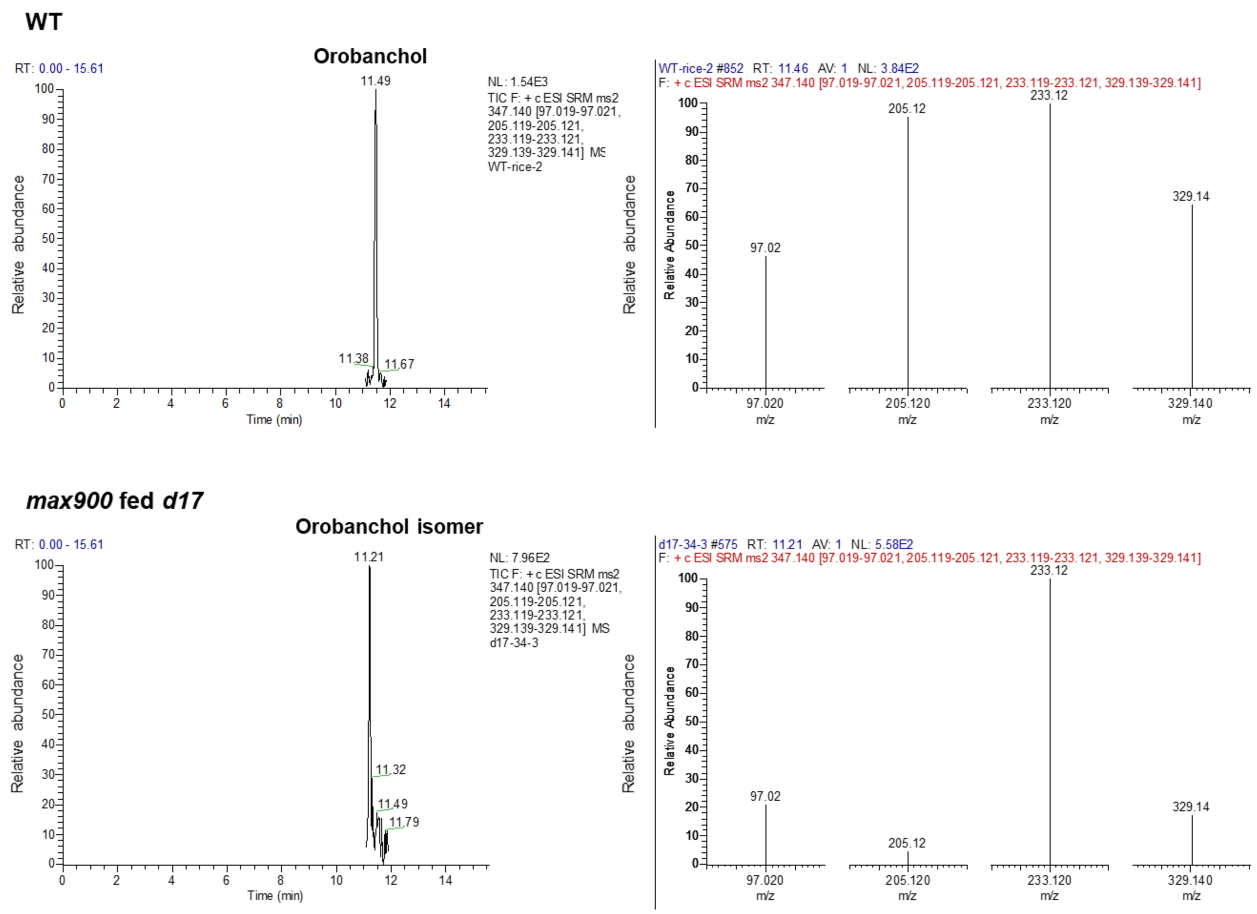

Supplementary Figure 8. Multiple reaction monitoring (MRM) chromatograms of orobanchol and orobanchol isomer. The left-side panel indicated the retention time and the right-side panel showed the MRM of four m/z fragmentations with the diagnostic m/z 97.02 (D-ring).

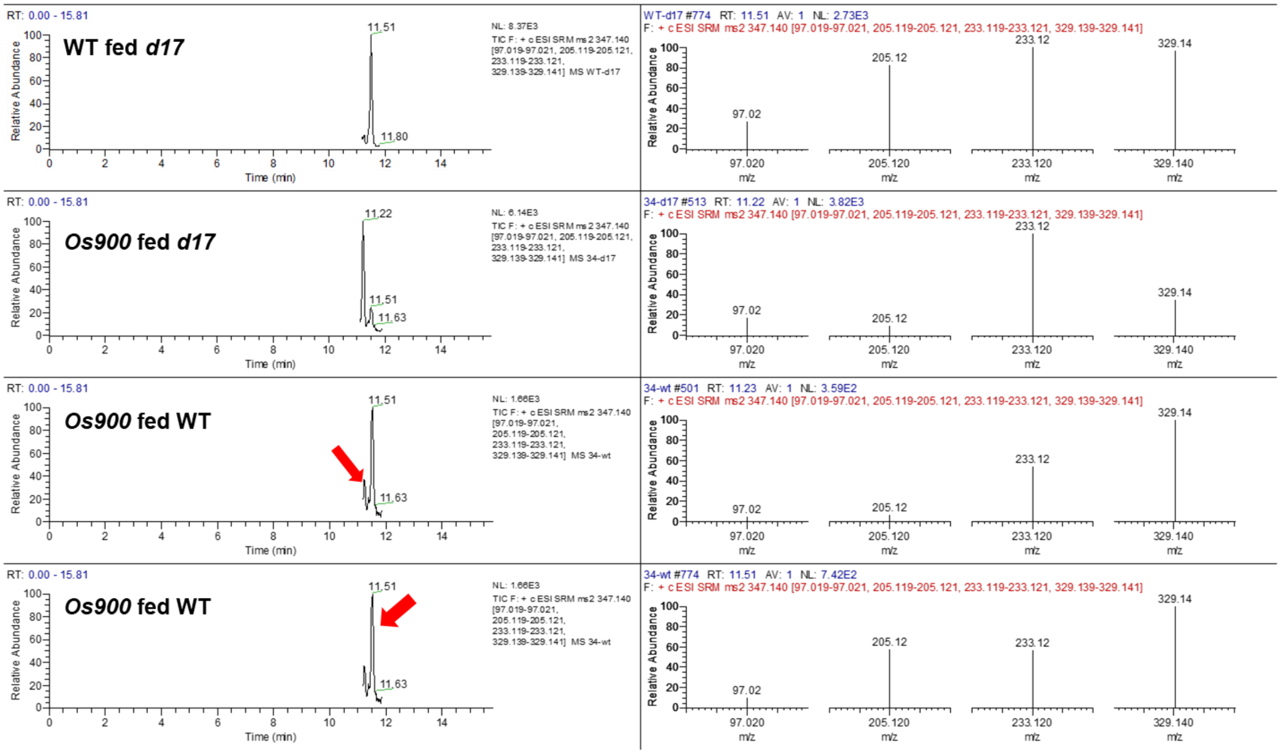

Supplementary Figure 9. Multiple reaction monitoring (MRM) chromatograms of orobanchol and orobanchol isomer after feeding experiment. Root exudate analysis after feeding *d17* with readjusted (Pi and pH) WT and *Os900* root exudates (WT /*Os900* fed *d17*) and WT readjusted (Pi and pH) with *Os900* root exudates. The left-side panel indicated the retention time and the right-side panel showed the ion-pairs. Red arrows indicate the elution peaks of orobanchol isomer with a retention time of 11.22 min while the retention time of orobanchol was 11.51 min.

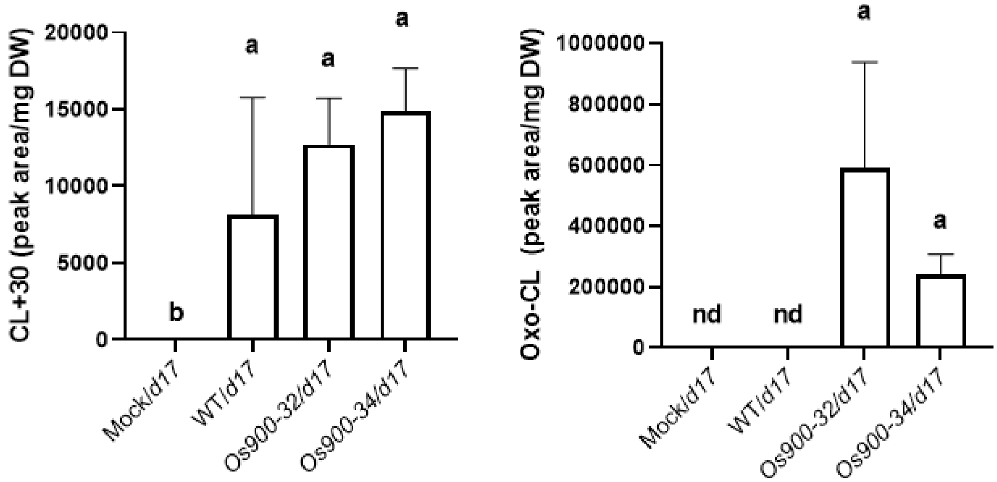

Supplementary Figure 10. SL analysis in rice roots. Identification and quantification of SLs in *d17* root tissues after feeding with mock (Mock/*d17*), WT (WT/*d17*) and *Os900*-KO lines (*Os900-32*/*d17* and *Os900-34*/*d17*) root exudates. The data are the mean ± SD number of samples n (3 ≤ n ≤ 5). The statistical significance is determined by one-way ANOVA and Tukey’s multiple comparison test.

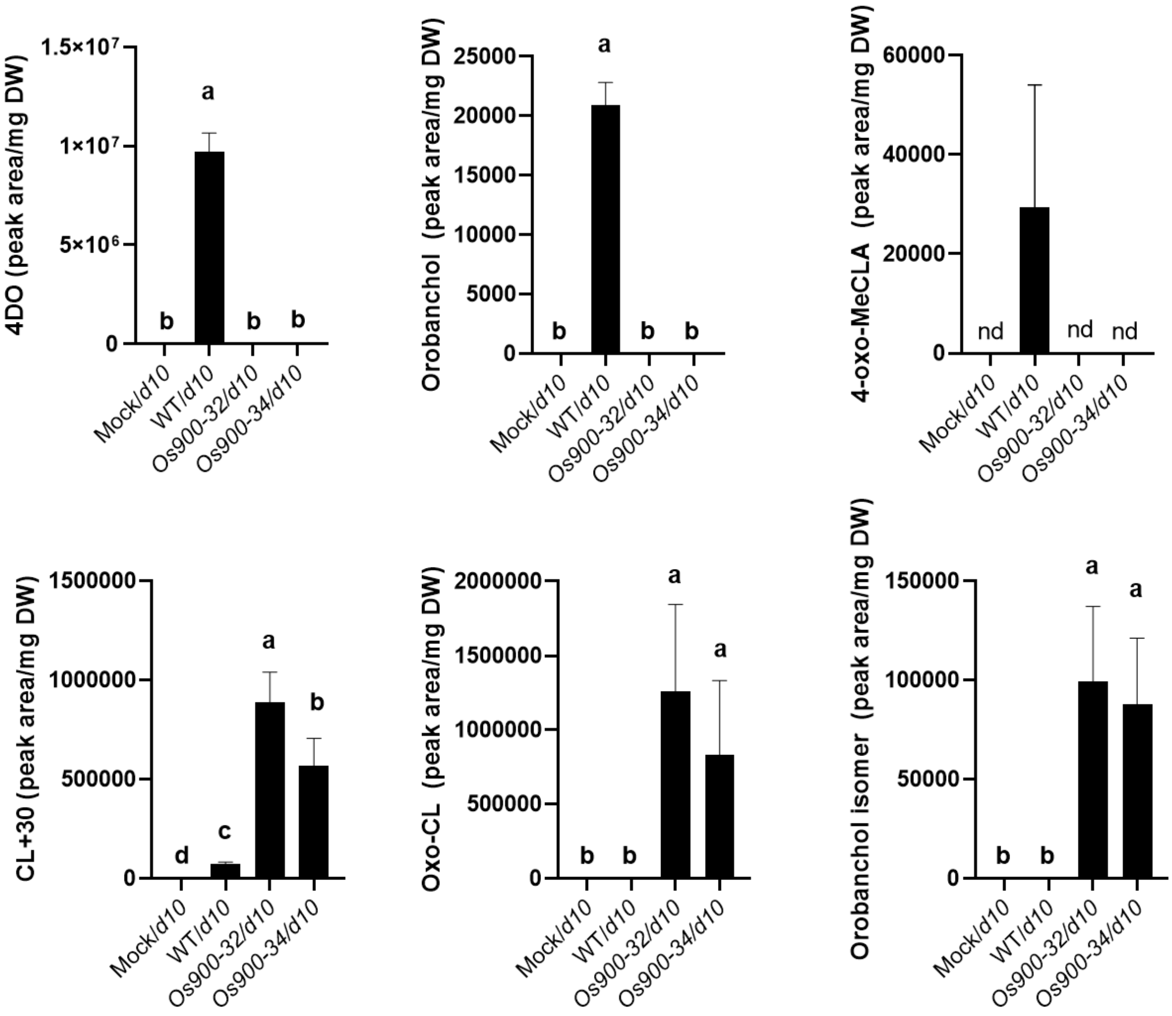

Supplementary Figure 11. Feeding experiments of *d10* seedlings using readjusted (Pi and pH) WT, *Os900-32* and *-34* root exudates. *d10* feeding experiment using root exudates of WT, *Os900-32* and *-34*. Different SLs – 4DO, orobanchol, CL+30, orobanchol isomer and oxo-CL – were quantified in root exudates of *d10* grown hydroponically under control conditions (Mock/*d10*) and using WT (WT/*d10*), *Os900-32* (*Os900-32/d10*) and *Os900-34* (*Os900-34/d10*) root exudates. The data are represented as the mean ± SD for a number of 6 replicates. The statistical significance is determined by one-way ANOVA and Tukey's multiple comparison test.

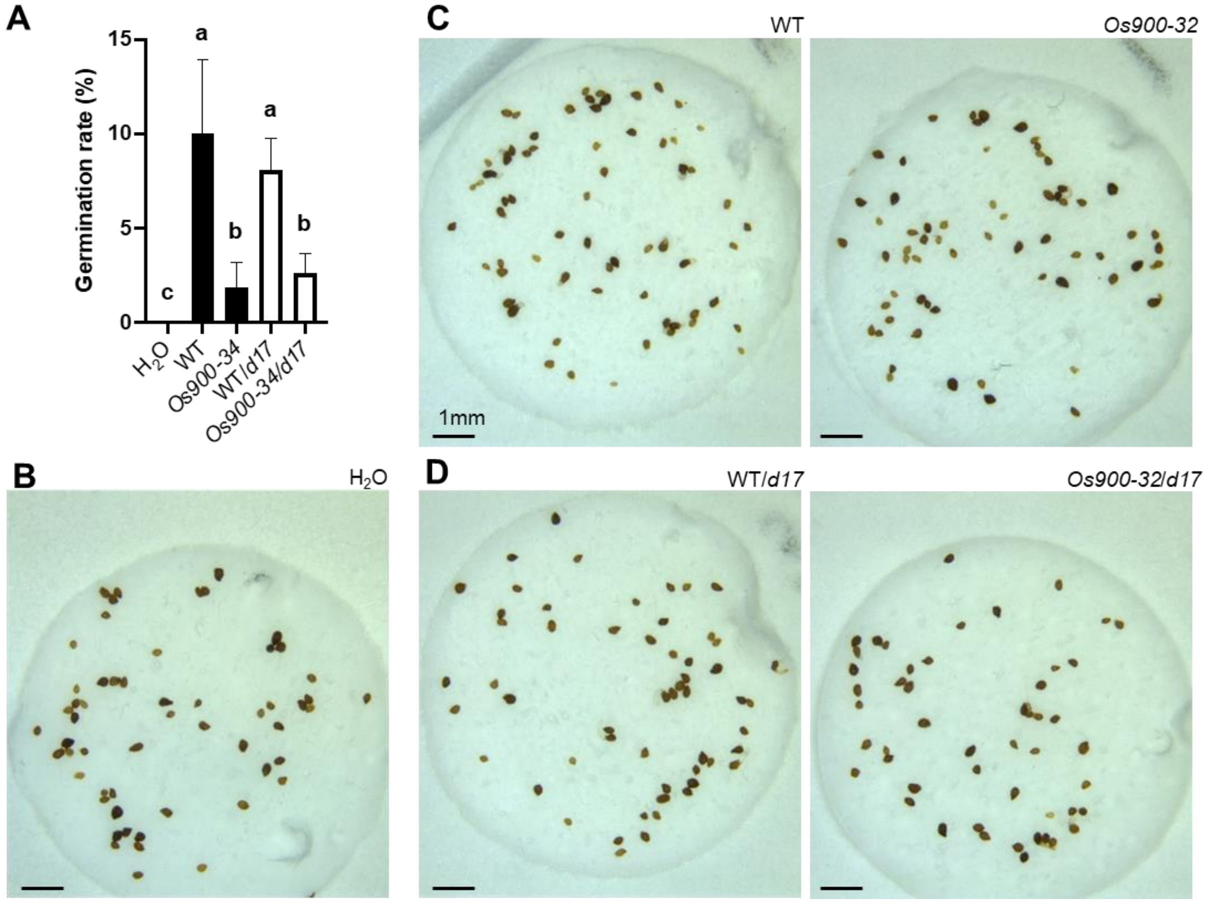

Supplementary Figure 12. Testing the germination potential of *d17* exudates after being fed with WT and *Os900-34* root exudates on *Phelipanche ramosa*. (**A**) *Phelipanche ramosa* germination rate in presence of water (H_2O), WT, *Os900-34* exudates as well as *d17* exudates after being fed with WT and *Os900-34* exudates (WT/*d17* and *Os900-34/d17*). The data are represented as the mean ± SD for a number of 3 replicates. The statistical significance is determined by one-way ANOVA and Turkey's multiple comparison test. Images of discs containing *Ph. ramosa* seeds under the different treatments: **B**- water (H2O), **C**- WT (left) and *Os900-32* (right) root exudates, **D**- root exudates of *d17* plants after being fed with WT (left) or *Os900-32* (right) root exudates. Scale bar=1mm

Table S1: Primers list for RT-qPCR

Primer name	Primer sequence	Organism
UbiQ	F:GCCCAAGAAGAAGATCAAGAAC<br>R: AGATAACAACGGAAGCATAAAAGTC	Rice Nipponbare
OsMAX1-900	F:ATTGTCAGCGATCCACTTC<br>R:GCGCCGTTCTTGAAATTG	Rice Nipponbare
OsMAX1-1400	F:GGCAGGTGCTCAAGAGGATT<br>R:TTTTGTCCATCTGTCCCCCG	Rice Nipponbare
OsMAX1-1900	F:GTTCCCCATAGGCCACCTTC<br>R:GCATTGGCCACAATCACCAG	Rice Nipponbare
OsMAX1-5100	F:GTGATAAAGGAGGCGATGAG<br>R:CTTTGGGAGTGTGTAGCC	Rice Nipponbare
